# Developmental origin and clinical profile of siamese twins in a tertiary care setting

**DOI:** 10.6026/973206300214267

**Published:** 2025-12-15

**Authors:** Archana Tiwari, Bindu Singh, Akshay Nigam, Vishnu Kumar Gupta, Parul Nema

**Affiliations:** 1 Department of Anatomy, Venkateshwara Institute of Medical Sciences, Gajraula, Uttar Pradesh, India; 2 Department of Anatomy, Baba Raghav Das Medical College, Gorakhpur, Uttar Pradesh, India; 3 Department of Radiotherapy, Gajra Raja Medical College, Gwalior, Madhya Pradesh, India; 4 Department of Community Medicine, SRVS Medical College, Shivpuri, Madhya Pradesh, India; 5 Department of Pathology, SRVS Medical College, Shivpuri, Madhya Pradesh, India

**Keywords:** Conjoined twins, embryology, thoracopagus, prenatal diagnosis, neonatal outcome, surgical separation

## Abstract

Conjoined twins represent a rare outcome of incomplete monozygotic embryonic division and are associated with high perinatal morbidity and mortality. Therefore, it is of interest to analyze 12 cases managed at a tertiary care center, focusing on developmental origin, clinical classification and perinatal outcomes. Thoracopagus was the most frequent type (41.7%) and prenatal diagnosis was achieved in 75% of cases, primarily through ultrasonography. Cesarean delivery was performed in most cases and neonatal survival was 41.7%, with successful surgical separation in two instances. Early diagnosis, precise anatomical delineation and multidisciplinary planning are critical for optimizing outcomes in these complex cases.

## Background:

Conjoined twins, also known as Siamese twins, represent a rare form of monozygotic twinning arising from incomplete embryonic division during early development [1, 2]. The phenomenon is estimated to occur in approximately 1 in 50,000 to 200,000 live births, with a notably high rate of stillbirth and early neonatal mortality [1, 3]. Female twins predominate in reported cases, accounting for up to three-quarters of live births [3]. Embryologically, conjoined twins result from a single zygote that invades a late separation process-typically around day's 13-14 post-fertilization-whens the primitive streak is forming, leading to variable fusion patterns depending on the site and timing of incomplete cleavage [4, 1]. The classification system traditionally identifies several types: thoracopagus (chest), omphalopagus (abdomen), pygopagus (sacrum), ischiopagus (pelvis) and craniopagus (skull), with thoracopagus being most prevalent [1, 3]. These classifications are of major clinical importance due to differing risks of shared organs and the complexity of separation procedures [3]. Approximately 75% of conjoined twins are joined at the thorax and/or abdomen-usually involving cardiac and hepatic sharing-while more specialized forms such as craniopagus are considerably rarer [1]. Associated structural anomalies, particularly cardiovascular and neural defects, are frequent and are often the primary determinants of prognosis and operative feasibility [3, 5]. Advances in prenatal imaging and detailed anatomical assessment through MRI have enabled early detection, multidisciplinary planning and optimized delivery strategy [1, 6]. Despite technological progress, prognosis remains poor: over half of diagnosed cases are stillborn or die shortly after birth and only a minority proceeds to successful surgical separation [3]. Furthermore, ethical, maternal and psychosocial considerations play a critical role in decision-making around continuation of pregnancy, delivery methods, postnatal interventions and long-term care. Therefore, it is of clinical and academic interest to describe the developmental origin, classification, associated anomalies and perinatal outcomes of conjoined twins managed at our tertiary referral center.

## Materials and Methods:

## Study duration and population:

The retrospective study included all cases of conjoined twins identified through labor and delivery records, neonatal ICU registers, prenatal ultrasound archives and operative logs. A total of 12 conjoined twin cases were included.

## Inclusion and exclusion criteria:

All prenatally or postnatally diagnosed cases of conjoined twins delivered at the institution were included. Cases with incomplete records or unclear diagnosis were excluded from final analysis.

## Data collection:

Relevant data were extracted from maternal records, prenatal imaging reports, operative notes, neonatal outcome sheets and pediatric surgical evaluations. Parameters collected included:

[1] Maternal details: age, parity, consanguinity, teratogenic exposure, antenatal care history

[2] Prenatal findings: gestational age at diagnosis, type of imaging used (ultrasound, MRI) and type of conjunction

[3] Delivery characteristics: gestational age at delivery, mode of delivery

[4] Neonatal profile: sex distribution, combined birth weight, type of conjunction, associated anomalies, survival outcome and surgical separation status

Conjoined twins were classified according to anatomical site of fusion (thoracopagus, omphalopagus, pygopagus, ischiopagus, craniopagus). Anomalies were recorded based on imaging, intraoperative findings and/or postmortem examination when available.

## Prenatal imaging protocol:

All antenatal scans were performed using high-resolution 2D ultrasonography, with supplementary fetal MRI in select cases to assess shared organ systems, particularly for thoraco-abdominal and craniopagus conjunctions. Diagnoses were confirmed by two independent radiologists.

## Outcome measures:

Primary outcome measures included frequency and type of conjoining, gestational age at diagnosis, survival status and feasibility of surgical separation. Secondary outcomes included associated anomalies and maternal factors.

## Data analysis:

Data were tabulated using Microsoft Excel and analyzed descriptively. Categorical variables were presented as frequencies and percentages; continuous variables as mean ± standard deviation.

## Results:

12 cases of conjoined twins were managed at our tertiary care center. Thoracopagus was identified as the most frequent form of conjunction, followed by omphalopagus and pygopagus, while rarer forms such as craniopagus and ischiopagus were also documented (Table 1 - see PDF). This distribution reflects global epidemiological trends, with thoracopagus commonly reported as the predominant type. Prenatal diagnosis was achieved in the majority of cases, with most detections occurring in the second trimester through routine ultrasonography. MRI was used selectively for further anatomical delineation in one-fourth of cases. The mean gestational age at delivery was preterm and cesarean section was the predominant mode of delivery (Table 2 - see PDF). Vaginal delivery was associated with poorer neonatal outcomes in this cohort. Analysis of neonatal sex distribution revealed a female preponderance and the average combined birth weight was within a viable range for surgical consideration. Discordant sex pairing was observed in a minority of cases (Table 3 - see PDF), which, while rare, can pose additional diagnostic challenges during early antenatal screening. Conjoined twins exhibited a range of structural anomalies based on the type of conjunction. Thoracopagus twins showed a high frequency of cardiac malformations, while omphalopagus twins frequently presented with shared liver and anterior abdominal wall defects. Neural and spinal defects were identified among craniopagus and pygopagus pairs, respectively (Table 4 - see PDF). These associations are critical for surgical planning and prognostication. Neonatal outcomes were poor in nearly 60% of cases, with one-third stillborn and an additional quarter succumbing within 48 hours of delivery. Of the five sets that survived the neonatal period, surgical separation was attempted in three, with successful outcomes achieved in two cases (Table 5 - see PDF). These findings highlight the high mortality associated with conjoined twinning and underscore the importance of early diagnosis and multidisciplinary planning. Maternal demographic analysis showed that the mean age was in the mid-20s, with no significant history of familial twinning or teratogenic exposures. Consanguinity was noted in a small proportion of cases (Table 6 - see PDF), although its relevance in conjoined twinning remains inconclusive due to the limited sample size.

## Discussion:

Conjoined twins represent a rare and challenging congenital anomaly, believed to result from incomplete division of a single fertilized ovum during the third week of gestation [1, 2 and 7]. The estimated incidence varies from 1 in 50,000 to 1 in 200,000 live births, with a higher prevalence in females and a significant proportion resulting in stillbirth or early neonatal demise [1, 3 and 8]. In our series, the most frequently observed type was thoracopagus, followed by omphalopagus and pygopagus. This distribution mirrors global patterns, where thoracopagus accounts for approximately 40% of all cases [7]. The high rate of prenatal diagnosis (75%) observed in this study reflects the increasing availability and accuracy of second-trimester ultrasonography. In selected cases, fetal MRI was employed to delineate the extent of visceral sharing, particularly in thoracoabdominal and craniopagus twins. Early diagnosis is crucial not only for parental counseling but also for delivery planning, given that vaginal delivery is often contraindicated due to the risk of obstructed labor and neonatal trauma [9]. In our cohort, cesarean section was the preferred mode of delivery in 75% of cases, consistent with recommended obstetric practice [10]. Survival in conjoined twins depends largely on the type of conjunction and extent of shared vital organs. In our series, 41.7% of twin pairs survived the neonatal period and surgical separation was attempted in three cases with two successful outcomes. This aligns with previously reported survival rates, which suggest that 25-50% of live born conjoined twins may be candidates for surgical separation depending on anatomical feasibility [11, 12]. Thoracopagus twins were most frequently associated with cardiac anomalies, while omphalopagus twins presented with shared abdominal viscera, including omphalocele and fused livers. Craniopagus and pygopagus cases exhibited central nervous system or spinal anomalies. These findings are consistent with previous anatomical and surgical studies, which highlight the significant challenges posed by shared cardiopulmonary or neurological structures [7, 12]. The observed female predominance in our study (58.3%) is also consistent with the well-documented gender bias in conjoined twinning, though the biological basis for this remains unclear [8]. Discordant sex pairing, which occurred in 16.7% of cases, is extremely rare and raises questions regarding early embryologic influences, possibly linked to chimerism or undetected anomalies in zygotic division [13]. Maternal characteristics such as young age, absence of prior anomalies and no significant exposure to teratogens suggest that environmental contributions to conjoined twinning are limited or sporadic. Consanguinity was present in a small proportion of cases (16.7%), similar to prior reports, but no direct causal link has been established [11]. In line with our findings, a recent case report of a vaginal delivery of dithoracic parapagus twins [14] underscores that, despite the predominance of cesarean delivery, management strategies must remain flexible to unanticipated presentations and tailored to anatomical variations of conjoined twins. This study is limited by its retrospective design and relatively small sample size, which is inherent to the rarity of the condition. Nonetheless, the findings contribute valuable insights into the clinical spectrum, prenatal detection and surgical outcomes of conjoined twins in a tertiary care setting.

## Conclusion:

Thoracopagus was the most common type of conjoined twinning observed in this study, with limited neonatal survival despite optimal perinatal management. Early prenatal diagnosis through ultrasonography and MRI facilitated timely intervention and delivery planning. Surgical separation was feasible in select cases, emphasizing the importance of individualized multidisciplinary evaluation based on the extent of organ sharing.
